# Vapor Phosphorylation of Cellulose by Phosphorus Trichlo-Ride: Selective Phosphorylation of 6-Hydroxyl Function—The Synthesis of New Antimicrobial Cellulose 6-Phosphate(III)-Copper Complexes

**DOI:** 10.3390/antibiotics10020203

**Published:** 2021-02-19

**Authors:** Marcin H. Kudzin, Zdzisława Mrozińska, Paweł Urbaniak

**Affiliations:** 1 Łukasiewicz Research Network—Textile Research Institute, Brzezinska 5/15, 92-103 Lodz, Poland; zmrozinska@iw.lodz.pl; 2 Faculty of Chemistry, University of Lodz, Tamka 12, 90-136 Lodz, Poland; pawel.urbaniak@chemia.uni.lodz.pl

**Keywords:** cellulose, phosphorylation, cellulose-*O*-hydrogenphosphates (III), cellulose-*O*-phosphates (III), abbreviations system, copper complexes, antibacterial activity, polymer functionalization

## Abstract

This research is focused on a synthesis of copper-cellulose phosphates antimicrobial complexes. Vapor-phase phosphorylations of cellulose were achieved by exposing microcrystalline cellulose to phosphorus trichloride (PCl_3_) vapors. The cellulose-*O*-dichlorophosphines (Cell-*O*-PCl_2_) formed were hydrolyzed to cellulose-*O*-hydrogenphosphate (P(III)) (Cell-*O*-P(O)(H)(OH)), which, in turn, were converted into corresponding copper(II) complexes (Cell-*O*-P(O)(H)(OH)∙Cu^2+^). The analysis of the complexes Cell-*O*-P(O)(H)(OH)∙Cu^2+^ covered: scanning electron microscopy (SEM), attenuated total reflection Fourier transform infrared spectroscopy (ATR-FTIR), atomic absorption spectrometry with flame excitation (FAAS), and bioactivity tests against representative Gram-negative bacteria (*Escherichia coli*) and Gram-positive bacteria (*Staphylococcus aureus*). The antimicrobial tests of synthesized Cell-*O*-P(O)(H)(OH)∙Cu^2+^ revealed their potential applications as an antibacterial material.

## 1. Introduction

Cellulose is an important structural component of the primary cell wall of green plants and it presents the most abundant organic polymer on Earth [1,2]. Many properties of cellulose depend on its chain length, a topology, and a surface state of the fibre [2,3,4]. 

The hydroxyl groups of cellulose can be partially or fully reacted with various reagents, including the coupling with acids and anhydrides, the grafting with siloxanes, isocyanates, and the grafting via free-radical initiation or ring opening polymerization, etc., affording various surface modified products [2,5,6,7,8,9,10,11,12]. 

**Figure 1:**
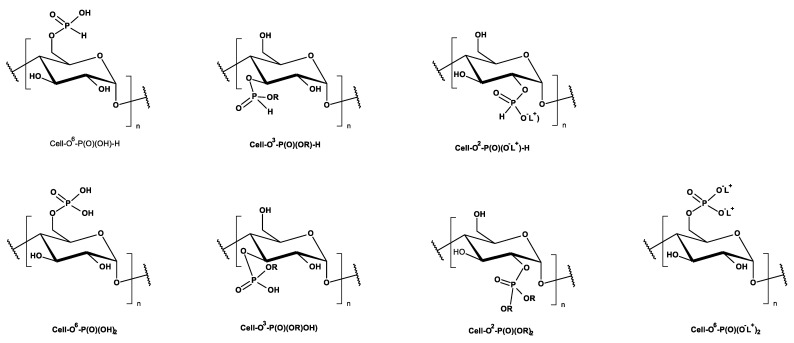
Major types of cellulose phosphates (III/V), derived from cellulose and phosphoric (III/V) acids (R = alkyl, aryl; L^+^ = cations). Position of phosphorylated HO group of cellulose is described using i-index at oxygen atom: as Cell-*O*^i^-phosphate group at i-atom of carbon ring (I = 2, 3, 6) and in the case of 6-phosphate Cell-O^6^-.

Another possibility of chemical modification of cellulose presents a phosphorylation [13]. Cellulose phosphates, more precisely named cellulose-*O*-phosphates (III or V) (synonyms: cellulose p; phosphocellulose, dihydrogen phosphate cellulose, cellulose, phosphate ester; phosphorylated cellulose), formed in the so-called cellulose phosphorylation reaction, have been used for decades e.g., sodium cellulose phosphate, under trade name calcibind in the treatment of calcium metabolism–related diseases, taking advantage of their high ability to bind calcium ions (e.g., [14,15,16]). Figure 1 presents the structures of various types of cellulose phosphoric (III/V) acids and corresponding phosphates (III/V).

Their chemistry has regularly been reviewed since the early decades of the 20th century, when they were first proposed as flame retardants [17,18]. Cellulose phosphorylation has also been applied in manufacture of cotton textiles (improvement of flame resistance, moderation of hydrophility-hydrophobity, etc.), cellulose-based nano-materials, ion adsorbents, and ion exchangers [19,20,21], etc.

As a matter of fact, phosphate cellulose [CAS Number: 9015-14-9] is manufactured by Sigma–Aldrich and recommended in protein chromatography [22] and ion exchange chromatography (e.g., [20,22,23]). 

Chemical modification of cellulose by phosphorylation also enhances its bioactivity (e.g., the treatment of calcium metabolism–related diseases) and it provides new derivatives and biomaterials with specific end uses (e.g., [24,25,26,27,28]). 

Therefore, the synthetic chemistry of this class of biomaterials has been developed for decades, affording a variety of synthetic procedures leading to cellulose-phosphates, in majority focused on cellulose-phosphates P(V) [13]. 

**Figure 2:**
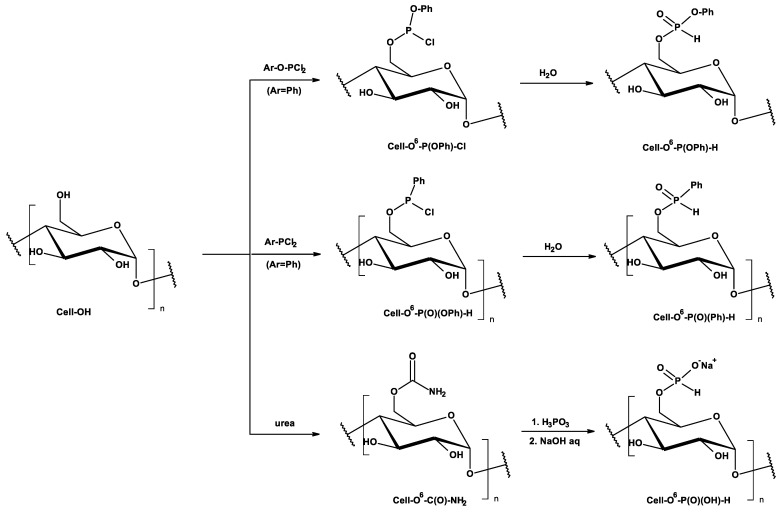
Major types of cellulose phosphorylations by P(III) reagents, assuming the formation of 6-esters of cellulose and phosphoric (III) acid—Cell-*O*^6^-P(O)(OH)-H (cellulose-*O^6^*-hydrogenphosphates (III), cellulose-*O^6^*-phosphates (III)) and/or esters of cellulose and aryloxy-phosphoric (III) acids (Ar-O-P(O)(OH)-H) - Cell-*O*^6^-P(O)(OAr)-H and C-phosphonic acids (Ar-P(O)(OH)-H) - Cell-*O*^6^-P(O)(Ar)-H. (In the path 3 the mixtures of Cell-O^2^-P(O)(OH)-H, Cell-*O*^3^-P(O)(OH)-H, and Cell-*O*^6^-P(O)(OH)-H were documented [33,34]).

The synthesis methods of cellulose-*O*-phosphates (P(III)) and generally applied P(III) reagents are presented in Figure 2 and characterized in Table 1 [29,30,31,32,33,34]. 

**Figure 3:**
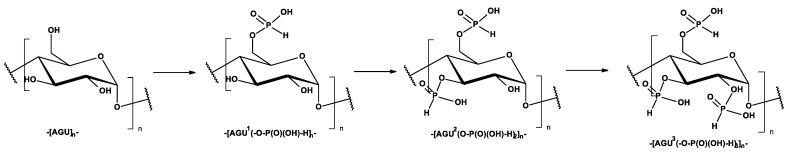
Gradual phosphorylation of cellulose (AGU^m^ = AGU units deprived m(OH) (*m* = 1–3) functions; ─[AGU^1^(-*O*-P(O)(OH)-H)]_n_─, DS = 1; ─[AGU^2^(-*O*-P(O)(OH)-H)_2_]_n_─, DS = 2; ─ [AGU^3^-(*O*-P(O)(OH)-H)_3_]_n_─, DS = 3).

These procedures afforded cellulose phosphates/cellulose phosphoric acids with differential phosphorus content, dependent on the applied conditions. Such phosphorylations occurred gradually step-by-step (-[AGU]_n_-)→(-[AGU-P(O)(OH)-H]_n_-)→(-[AGU-(P(O)(H)(OH))_2_-H]_n_)-→([-AGU-(P(O)(OH)-H)_3_]_n_-) (Figure 3), without/or with subsequent dissociation of molecular cellulose from cellulose microfibrils affording finally surface cellulose modified phosphates of cellulose phosphate molecular chains.

However, if the phosphorylation is carried out in mild conditions, only accessible hydroxyl groups are esterified; in other words, the cellulose microfibrils are only phosphorylated on the surface, with typical regioselectivity (primary 6-HO groups). Such conditions are fulfilled during vapor phase reactions. In this paper, we reveal our results on vapor phase phosphorylation of cellulose by means of PCl_3_ (Cell-OH→Cell-*O*-P(O)(OH)-H). 

**Figure 4:**
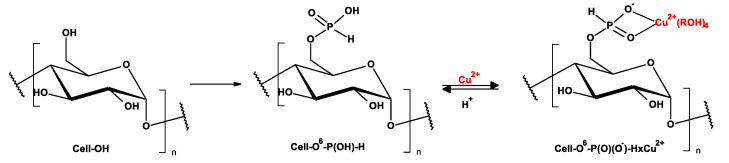
Synthesis of complexes of cellulose-*O*^6^-phosphates and copper ions (Cell-*O*^6^-P(O)(O^−^)-H→Cell-*O*^6^-P(O)(O^−^)-H × Cu^2+^) (ROH- water molecules or cellulose hydroxyls).

As a part of our research program directed on biologically active functionalized phosphonates [35,36] and their polymer hybrids [37,38], we present our results on PCl_3_ vapor phase phosphorylation of cellulose to cellulose-*O*-phosphates (III) (H-phosphonates) and their conversion into corresponding copper complexes [(Cell-OH→Cell-*O*-P(O)(OH)-H)→Cell-*O*-P(O)(O^−^)-H × Cu^2+^ (Figure 4)].

## 2. Results and Discussion

Physical chemistry of cellulose-phosphates (III and V) has been well documented in the literature [13], however in the majority concerning cellulose dihydrogen phosphates(V) Cell-*O*-P(O)(OH)_2_, and with much less dealing with cellulose hydrogen phosphates (III) Cell-*O*-P(O)(OH)-H and derivatives [29,30,31,32,33,34]. 

Cellulose hydrogen phosphates (III) Cell-*O*-P(O)(OH)-H, obtained by vapor phosphorylation of cellulose by means of PCl_3_ and subsequent work-up with water, further called cellulose phosphates (III), were characterized using ^31^P-NMR, scanning electron microscopy (SEM), FTIR, and potentiometric titration. Cellulose phosphates, on the basis of ^31^P-NMR–Cell-*O*^6^-P(O)(OH)-H, were also converted into copper complexes (Cell-*O*^6^-P(O)(OH)-H→Cell-*O*-P(O)(O^−^)-H × Cu^2+^), which were characterized using the atomic absorption spectrometry with flame excitation (FAAS) method for the determination of copper content. The formed Cell-*O*^6^-P(O)(O^−^)-H × Cu^2+^ samples were tested for their bioactivity testes against representative Gram-negative bacteria (*E. coli*) and Gram-positive bacteria (*S. aureus*).

### 2.1. Phosphorylation of Cellulose

**Figure 5:**
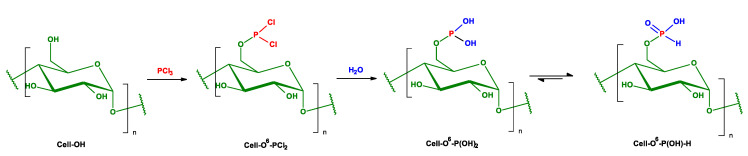
Chemical schemes of vapor phosphorylation of cellulose with PCl_3_.

The phosphorylation reactions of the cellulose in the exposure of phosphorus trichloride (PCl_3_) were carried out in the set consisting of two glass weighing bottles: the larger one (D vs. H: 40 mm × 40 mm) and the inner vessel (D vs. H: 20 mm × 20 mm) (the figure of the reaction vessel is given in the Supplementary part)). A 0.05 g portion of cellulose was poured into the inner vessel. Raschig rings were placed in the larger bottle (h = 1 cm), and then PCl_3_ (1 mL) was added, followed by placing the inner vessel (with cellulose) into the bottle with PCl_3_, followed by the hole closing with a lid. Figure 5 presents chemical schemes of vapor phosphorylation of cellulose.

The reactions were carried out for up to 72 h, after which the inner liner was removed from the reactor, the contents were flushed with nitrogen, and then placed in a beaker of water (25 mL). After 15 min., cellulose phosphate (P(III) (9 h) suspension is filtered on a Schott–Duran sintered disc filter funnel, washed on the filter with water (5 mL), and then transferred into a beaker (100 mL) with methanol (5 mL). The suspension was stirred by 5 min, again filtered on a Schott-Duran sintered disc filter funnel, and then dried in a vacuum desiccator over solid KOH for 24 h.

### 2.2. Complexation Reactions of Phosphorylated Cellulose—Synthesis of Complexes

The samples of phosphorylated cellulose (Cell-*O*^6^-P(O)(OH)-H), obtained after 6, 24, 48, and 72 h cellulose phosphorylation by PCl_3_, (determined further in the text as Cell-*O*^6^-P(O)(OH)-H(t), were *t* = 6, 24, 48 and 72 h) were mixed with a solution of copper(II) nitrate in HNO_3_ (Table 2) and stirred for 2 h, then the solution was filtered off, rinsed with water, dried to constant weight at 50 °C, and then transferred to a vacuum desiccator over KOH.

The ^31^P-NMR spectra of the cellulose-*O*-phosphate(III) (Cell-*O*^6^-P(O)(OH)-H) samples were recorded in the Bruker Avance III 600 spectrometer at frequency 242,9 MHz Elemental analyses (C and H) were recorded on an Elemental Analyzer Euro EA (Eurovector, Pavia, Italy).

### 2.3. Solubility of Cell-O^6^-P(O)(OH)-H

The solubility of the prepared sample would be the useful attribute in further derivatizations or potent applications. Generally, the solubilities of cellulose phosphates present scarcely explored field. Thus, Reid and Mozano [39] claimed that cellulose-*O*-phosphates cannot withstand the rigorous treatment of 6 N sodium hydroxide, but in ca. 1 N NaOH are solubilized during 1 h reflux temperature [39], but the cellulose triphosphates (DS = 2.9] swell considerably in water, forming a consistent translucent gel according to Granja [40].

Cellulose phosphates, obtained by molten urea-phosphoric (III/V) acids methods, are initially isolated by the dissolution of the reacted mixtures in 1 N aqueous sodium hydroxide and then precipitated with methanol (Cell-*O*-P(O)(OH)-H→Cell-*O*-P(O)(O^−^Na^+^)-H [32]; Cell-*O*-P(O)(OH)_2_→Cell-*O*-P(O)(O^−^Na^+^)_2_ [41]). In a procedure described by Suflet [32], this process was repeated three times, in order to re-move the residual reagents.

Cell-*O*-P(O)(OH)-H samples, obtained by Petreus [33], are white powders, insoluble in water, aqueous NaOH conc. solution, acetone, and DMF [33]. However, the Cell-*O*-P(O)(OH)-H sample (P = 13.4%; DS = 0.97) was dissolved in water, according to Petreus [33]. 

We assumed that ionic liquid based solvents that were applied for the dissolution of cellulose [42,43,44,45,46,47,48,49] can also be applied for the dissolution of cellulose-*O*-phosphates.

Table 3 provides the results of our investigations on the solubility of Cell-*O*^6^-P(O)(OH)-H.

### 2.4. ^31^P-NMR of Cell-O-P(O)(OH)-H

Cellulose and cellulose based polymers are usually analyzed/characterized using NMR solid state techniques [50,51,52] due to the insolubility of cellulose in a majority of common solvents [2,4,5,6].

Cellulose-*O*-phosphates (phosphorylated celluloses), due to a presence of phosphorous atom in molecules, have been analyzed by ^31^P-NMR in a majority in solid state mode [27,28,34,47,53,54,55,56,57,58,59,60,61]. Thus, in Gospodinova paper [34], ^31^P-NMR solid-state spectrum of Cell-*O*-P(O)(OH)-H (prepared by the phosphorylation of cellulose in molten urea-phosphorous acid mixture (DS = 0.2)) contained the signals in the 2.5–7.5 ppm region, corresponding to the three positions of substitution, namely a signal at 2.6 ppm assigned to P–O–C6, and the doublets at 5.1–5.2 ppm (P-O-C2) and at 7.5–7.6 ppm (P–O–C3) (Table 4).

**Figure 6:**
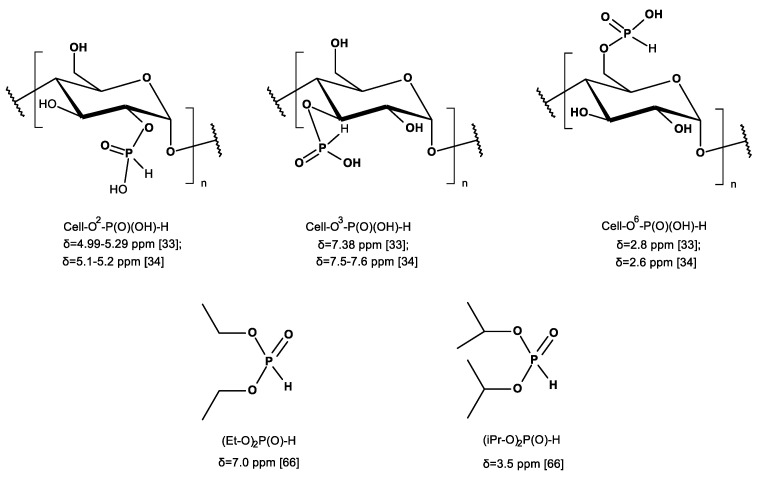
Structures of Cell-*O*^i^-P(OH)-H and representative dialkylphosphates (III) with primary and secondary alkoxyl, and corresponding literature ^31^P-NMR chemical shifts [ppm].

In the only paper of Petreus [33], the Cell-*O*-P(O)(OH)-H sample (prepared by phosphorylation of cellulose in molten urea-phosphorous acid mixture (P = 13.4%; DS = 0.97)) was dissolved in D_2_O and analyzed on a Avance III 400 spectrometer, operating at 161.97 MHz for ^31^P nuclei. ^31^P-NMR spectrum of this sample showed a set of thirteen peaks, with the main at 2.58 ppm and two doublets at 4.99–5.29 ppm and at 7.38 ppm, which were assigned by authors to P–O–C6, P–O–C2 and P–O–C3, respectively. All of the signals according to the Authors corresponded to monosubstituted phosphorous acid esters of cellulose. Figure 6 presents structures of Cell-*O*^i^-P(OH)-H (i = 2, 3, and 6) and representative dialkylphosphates (III) with primary and secondary alkoxyl, and corresponding ^31^P-NMR chemical shifts (δ [ppm]).

We used ^31^P-NMR solid state analysis because our Cell-*O*-P(O)(OH)-H sample has exhibited solubility neither in D_2_O nor in representative ionic liquids (e.g., TBAA). 

**Figure 7:**
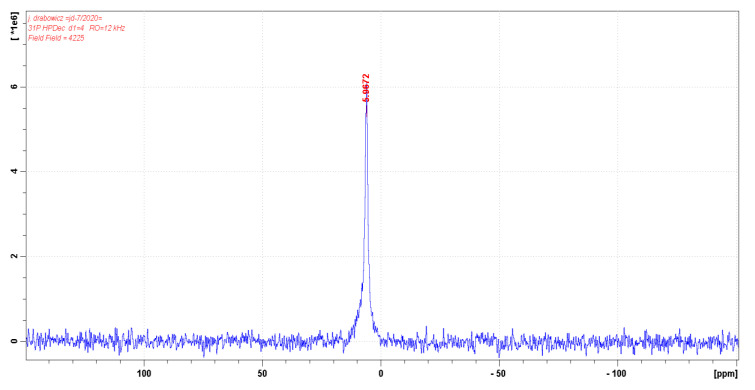
^31^P-NMR solid state spectrum of Cell-*O*-P(O)(OH)-H-48 sample.

**Figure 8:**
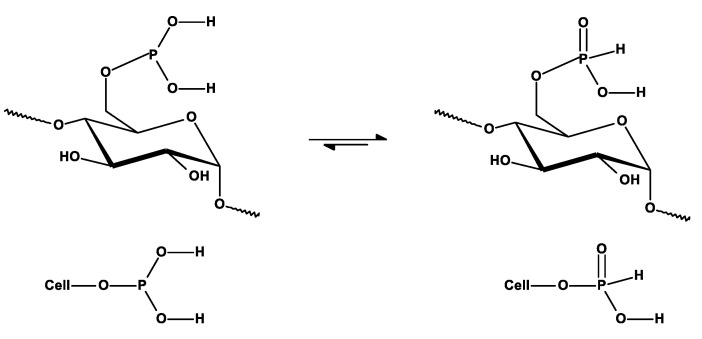
Equilibrium and structures of cellulose-*O*^6^-phosphate (III) (Cell-*O*^6^-P(OH)_2_⇌Cell-*O*-P(O)(OH)-H).

We assumed that, during the phosphorylation in mild conditions (as we applied), the formation of cellulose 6-phosphate(III) (Cell-*O*-P(O)(OH)-H) will be preferred due to the highest reactivity of 6-hydroxyl group of cellulose [62]. In Figure 7, the ^31^P-NMR spectrum of cellulose-*O*-phosphate (III) (Cell-*O*-P(O)(OH)-H) only exhibits one signal with chemical shift δ = 5.067 ppm, which we assigned to 6-phosphate(III) of cellulose (Cell-*O*^6^-P(O)(OH)-H), resulting from mild conditions of applied phosphorylation (see Table 1 for comparison). This signal, in contrary to earlier reports [33,34], we attached to 6-phosphate (III) structure, due to higher accessibility and reactivity of primary hydroxyl group in the phosphorylation [43], and, because of that, branching at the carbinol carbon C-C*(OH)-C of phosphate (C*-O-P(O)(OH)-H) usually affords upfield shifts of the phosphorous nuclei (e.g., diethyl H-phosphonate δ 7. Ppm, whereas di-isopropyl H-phosphonate δ 3.5 ppm) [62] (Table 4). Figure 8 presents the structures of cellulose 6-phosphate (III) (Cell-*O*^6^-P(O)(OH)-H).

### 2.5. SEM—Scanning Electron Microscopy of Cellulose Phosphates

SEM was employed to evaluate the morphological structures of the cellulose phosphates studied. Table 4 characterizes the morphology of various types of cellulose and their derivatives.

**Figure 9:**
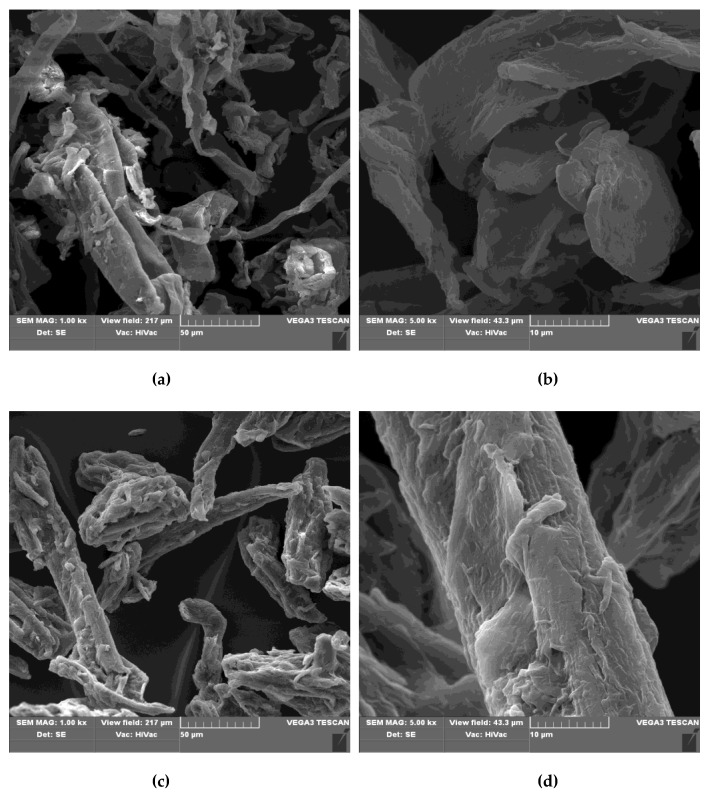

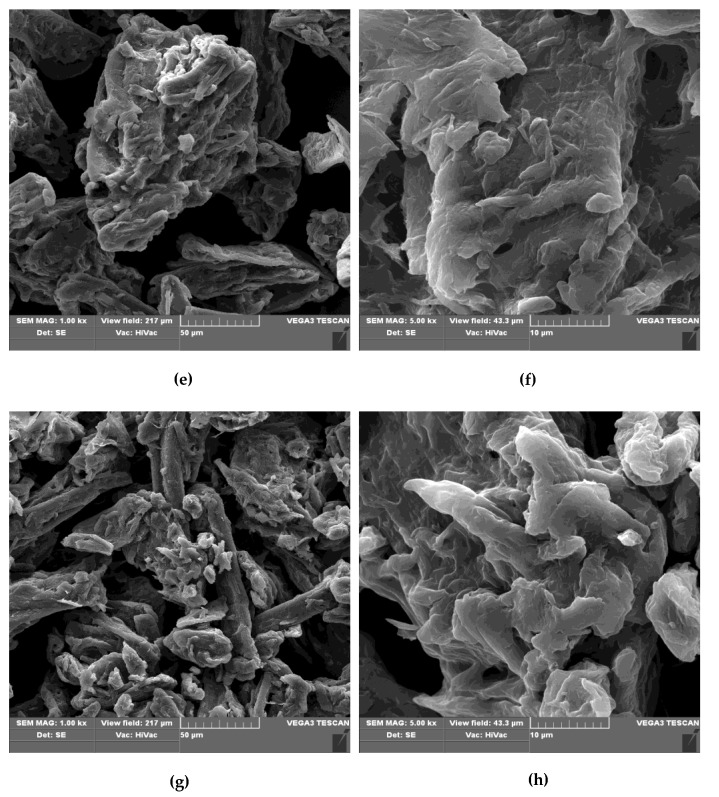
Scanning electron microscopy (SEM) micrographs of different magnifications (1 k and 5 k) for: (**a**,**b**) unmodified cellulose microcrystalline; (**c**,**d**) phosphorylated cellulose by PCl_3_ after 48 h reaction time and work-up (Cell-*O*^6^-P(O)(OH)-H(48 h)); (**e**,**f**) phosphorylated cellulose by PCl_3_ after 72 h reaction time and work-up (Cell-*O*^6^-P(O)(OH)-H(72 h)); and, (**g**,**h**) cellulosic Cu-complex (Cell-*O*^6^-P(O)(O^−^)-H × Cu^2+^ (48 h).

Figure 9 presents the SEM images ( ×1000 and ×5000 magnifications) of cellulose sample, phosphorylated derivatives Cell-*O*^6^-P(O)(OH)-H, and Cu-complex Cell-*O*^6^-P(O)(OH)-H × Cu^2+^.

**Table 4:** Morphology of various cellulose types and their derivatives.

No.	Fibre	Characterization	SEM [Image Magnification]	Ref
1	MCC (Avicel PH-101)	Nonfibrous nature and the presence of pinholes at its surface.	×1000; ×5000	[63]
	CNF& Ac-CNF	Cellulose nanofibers and acetylated nanofibers	×15,000	[64]
	BC	Interwoven mesh of BC fibrils network; The average fibril diameter 71 nm	×5000	[57]
	BCC5	Interwoven mesh of BC fibrils network; The average fibril diameter 107 nm		
2	MCC	Rough surface morphology	×1000	[58]
	MCC-P	Sponge-like surface character and compact structure		
3	CNF	Cellulose nano fibers: diameter 0.5–1.0 µm	×20,000	[59]
	CNF-P			
	CNF/HAp			
4	BC	Cellulose nano fibers: diameter 2.25 µm	×20,000	[60]
	BC-P			
	BC-P/TiO_2_			
5	KF	Cellulose fibers: diameter 20–25 µm	×1000; ×2000	[61]
	KF-P	Cellulose fibers with holes; diameter 20–25 µm;		

Ac-CNF—Acylated Cellulose NanoFibers; BC—Bacterial Cellulose; BC-P—Bacterial Cellulose Phosphate; CNF—Cellulose NanoFibres; CNF-P—Cellulose NanoFibres Phosphate; BCC5—Bacterial Cellulose-Chitosan (95:5); HAp—hydroxyapatite; KF—Kraft Fibres; KF-P—Kraft Fibres Phosphates; MCC—MicroCristalline Cellulose; MCC-P—MicroCristalline Cellulose Phosphate.

The presented micrographs do not exhibit substantial morphological changes that are caused by the successive derivatization of cellulose, namely Cell-OH (Figure 9a,b)→Cell-*O*^6^-P(O)(OH)-H (Figure 9c–f)→Cell-*O*^6^-P(O)(O^−^)-H × Cu^2+^ (Figure 9g,h), in spite of structural changes caused during the phosphorylation and subsequent complexation. This fact can result from the following reasons:

(a) the phosphorylation occurs on the surface HO-C6 group of cellulose and, therefore, does not disturb hydrogen bonds formed between adjacent cellulose chains in the starting cellulose;
(b) the phosphorylation causes the substitution the polar HO group by even more polar -P(O)(OH)-H group with two groups able to form hydrogen bonds; and,
(c) the phosphorylation takes place in ca. 2 AGU subunits in (AGU) 100 chains (DP = 0.018).

Similarly, the formation of copper complex (Cell-*O*^6^-P(O)(OH)-H(48 h)→Cell-*O*^6^-P(O)(O^−^)-H(48 h) × Cu^2+^) does not accompany substantial changes of the morphology, presumable for the reasons cited above. 

A similar phenomenon was described by Keshk [65]. They observed that the microstructures of structurally different compounds, namely: starting cellulose 6-phosphate (DP = 1), cellulose-6-phosphate 2,3-dialdehyde, and corresponding cellulose-6-phosphate 2,3-diimines, analyzed by SEM, did not exhibit significant changes at (1 k× and 5 k× magnifications). 

### 2.6. Attenuated Total Reflection Fourier Transform Infrared (ATR-FTIR) Spectroscopy

Mid-infrared and Raman spectroscopy are versatile tools in the characterization of structural modifications of biomolecules, being complementary techniques for their structural analysis [66] in these structural analysis of various cellulose-*O*-phosphates ([67] and Table 5 and Table 6).

**Figure 10:**
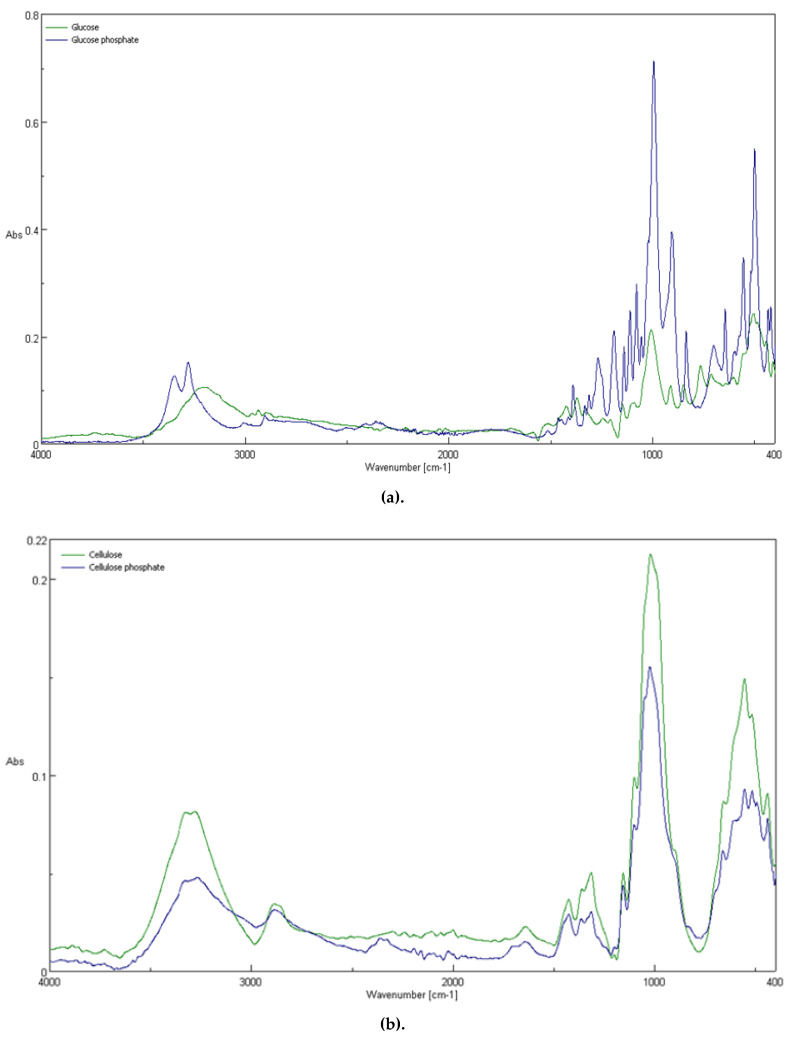
Comparison of attenuated total reflection Fourier transform infrared (ATR-FTIR) spectra of: (**a**) glucose and glucose phosphate (V) (Sigma–Aldrich); (**b**) cellulose (Avicel) and cellulose-*O*-phosphate(V) Cell-*O*-P(O)(OH)_2_ (Sigma-Aldrich).

**Figure 11:**
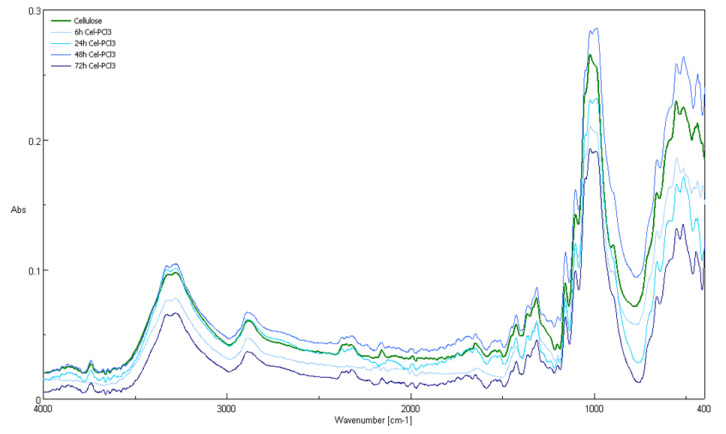
ATR-FTIR spectra of unmodified cellulose and cellulose samples formed during 6 h, 24 h, 48 h, and 72 h of vapour phosphorylation of cellulose by PCl_3_ (Cell-*O*^6^-P(O)(OH)(h)).

The FT-IR spectroscopy was used in this work for the study of the chemical structures of the fibers after chemical modification. Figure 10 and Figure 11 show ATR-FTIR spectra of: unmodified cellulose; Cell-*O*^6^-P(O)(H)OH sample—obtained by 48 h exposition of cellulose in PCl_3_ vapors; cellulose-*O*-phosphate(V) Cell-*O*-P(O)(OH)_2_ (Sigma-Aldrich) and D-Glucose 6-phosphate sodium salt. An ATR-FTIR spectrum of unmodified cellulose, contains bands, which, according to Tasker et al. [68], can be assigned, as follows: 670 cm^−1^ (OH wagging), 893 cm^−1^ (C_1_ group vibration), 1000 cm^−1^ (C-C stretching modes), 1060 cm^−1^ (C-C-O stretching mode), 1120 cm^−1^ (C-O-C asymmetric stretch), 1370 cm^−1^ (CH_2_ bending mode), 1429 cm^−1^ (in-plane OH bend), 2893 cm^−1^ (C-H stretching mode), and 3300 cm^−1^ (intermolecularly bonded OH stretching mode).

**Table 6:** Characterization of FTIR spectra of glucose, cellulose, glucose-phosphate, and cellulose-phosphates.

Compound/Frequency [cm^−1^].					Type of Vibrations
Gluc-OH ^/a^	Gluc-*O*^6^-P(O)(OH)_2_	Cellulose (Avicell)	Cell-*O*^6^-P(O)(OH)-H	Cellulose-*O*-P(O)(OH)_2_	
3410, 3333	3360	3300	3300	3300	intermolecularly bonded OH stretching mode
2944, 2913	2930	2893	2893	2893	C-H stretching
	2860				symmetric vibration of C-H
			2320		P-H
1849 to 1634					Vibrations of C=O
1450	1470				bending vibration of CH
1362 to 1191	1380	1429	1429	1429	in-plane OH bend
		1370	1370	1370	CH_2_ bending mode
	1250–1300		1250–1300	1250–1300	P=O
		1120	1120	1120	C-O-C asymmetric stretch
		1060	1060	1060	C-C-O stretching mode
1191 to 995		1000	1000	1000	C-C stretching modes
		893	893	893	C_1_ group vibration
		670	670	670	OH wagging
			520–600		P(O)-H

^/a^ Assignment according to Ibrahim et al. [69]. α-d-Glucose—Gluc-OH; Gluc-*O*^6^-P(O)(OH)_2_—Glucose-*O*^6^-phosphate. Vibrations derived from phosphoric(III/V) functions are marked in red.

A comparison of the FTIR spectra revealed that, for Cell-O-P(O)(X)OH, the appearance of a new band, at 2400 cm^−1^, was absent in the matter cellulose. There is a rather intense band at 1725 cm^−1^ that is not present in the spectrum of the original cellulose.

### 2.7. Alkalimetric Titration

**Figure 12:**
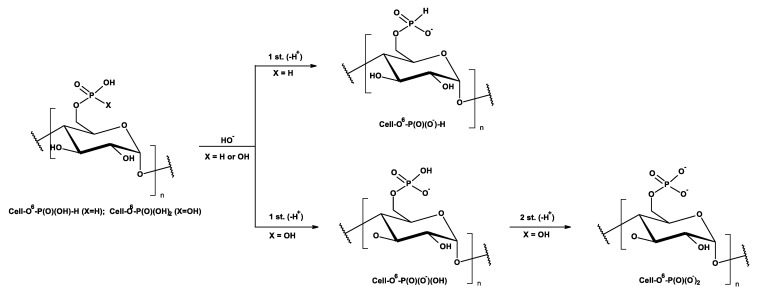
Schemes of alkalimetric titration of cellulose phosphoric acids.

Because of shapes of the titration curves of Cell-*O*^6^-P(O)(OH)-H and Cell-*O*^6^-P(O)(OH)_2_, resulted from one- or two-proton dissociation in reaction with hydroxide anion (Figure 12), such titration allows the identification, estimation, or semi-quantification of phosphoric groups in cellulose phosphoric acids (Table 7).

**Figure 13:**
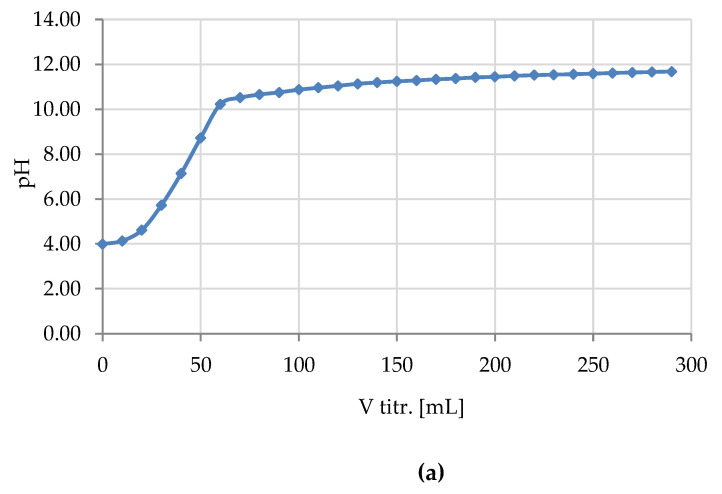

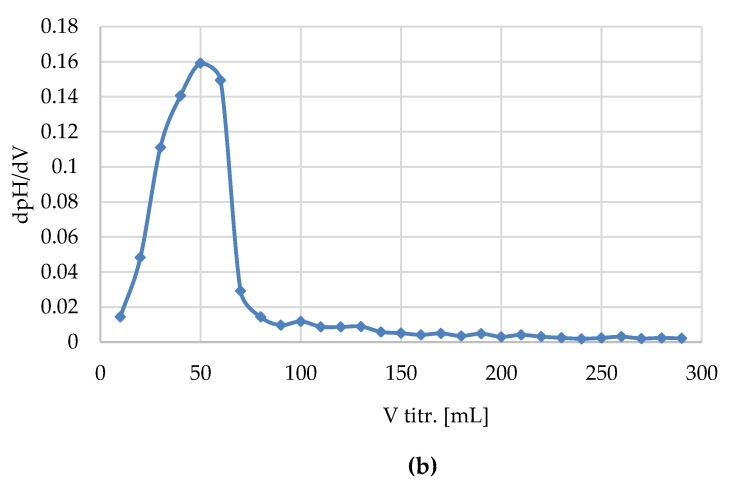
Titration curve of Cell-*O*^6^-P(O)(OH)-H(48 h) by 0.016 M KOH: (**a**) pH vs. V; (**b**) dpH/dV vs. V.

We carried out the direct titration of the sample of Cell-*O*^6^-P(O)(OH)-H, synthesized, in order to confirm the nature of phosphate function introduced into cellulose molecule by phosphorylation. Figure 13 presents the figure of the titration curve. 

One deflection point of the titration curve of Cell-*O*^6^-P(O)(H)(OH)-48 sample by KOH confirms the presence of the phosphate(P(III)) function in the molecule of phosphorylated cellulose and the absence of the corresponding phosphate(P(V)), excluding its oxidation (Cell-*O*^6^-P(O)(OH)-H→Cell-*O*^6^-P(O)(OH)_2_).

Elemental analyses of prepared cellulose-*O^6^*-phosphate (III) (cellulose-*O^6^*-phosphoric (III) acids) samples were accomplished while using combustion analysis (Elemental Analysis) and Inductively Coupled Plasma-Mass Spectrometry (ICP-MS). Table 8 summarizes the results.

The Degree of Phosphorylation/Substitution of cellulose values were calculated while using the following Equation (1) [33]:

(1) $$\mathrm{DP}=\frac{162.1\cdot P\left(\%\right)}{3100-64\cdot P\left(\%\right)}$$

where 162.1 is the molar mass of AGU (anhydro-glucose unit); %P is the percentage of phosphorus content in cellulose phosphates. 

**Figure 14:**
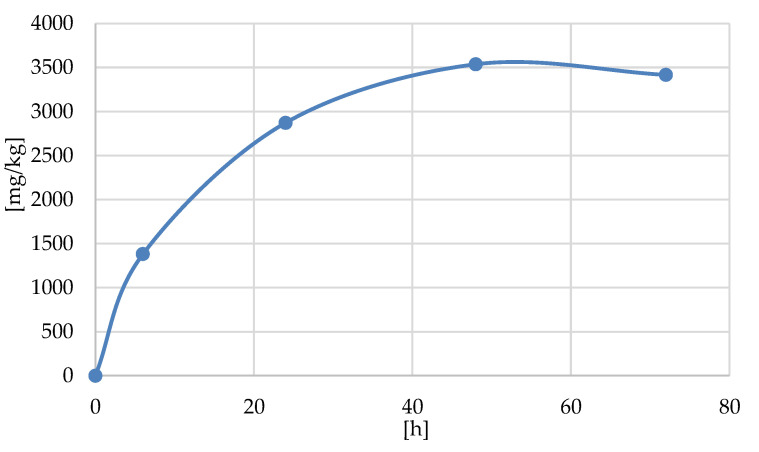
The course of phosphorylation of cellulose by PCl_3_ (Cell-OH→Cell-*O*^6^-PCl_2_→Cell-*O*^6^-P(O)(OH)-H) obtained on the basis of phosphorus determination in phosphorylated samples Cell-*O*^6^-P(O)(OH)-H(t[h]).

Cellulose phosphorylation, which was carried out in heterogeneous conditions, should lead to the anisotropic distribution of phosphoryl groups (P(III): -O-P(O)(H)(OH) between surface and bulk, due to the uneven accessibility of the fiber wall. The obtained results graphically illustrated in Figure 14, namely the nearly linear increase of phosphorus content in reaction time 0–24 h and slow decrease in the range 24–48 h with the plateau in the range 48–72 h, suggest that the vapor phosphorylation occurs mainly at the cellulose surface (with DP up to 0.0185 ± 0.0005). These results were confirmed by EDS determination of phosphorous, showing a similar shape of the curve with the plateau in the range of 48–72 h. 

The supplemental results on ^31^P-NMR (Figure 6) and alkalimetric titration (Figure 13) confirm the selective monophosphorylation of 6-hydroxyl group of cellulose. 

### 2.8. Digestion of Samples Prior to Phosphorus and/or Copper Determination

**Figure 15:**
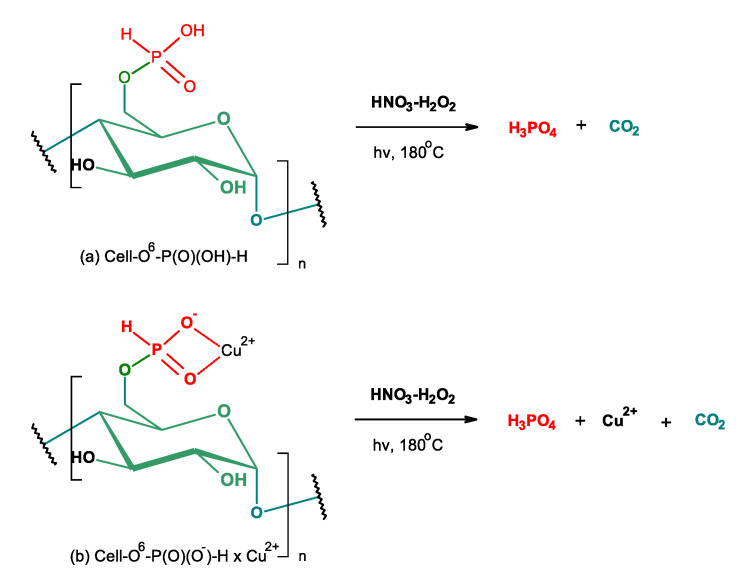
Digestion of (**a**) Cell-*O*^6^-P(O)(OH)-H and (**b**) Cell-*O*^6^-P(O)(O^−^)-H × Cu^2+^.

Cell-*O*^6^-P(O)(OH)-H and/or Cell-*O*^6^-P(O)(O^−^)-H × Cu^2+^ samples were degraded by wet digestion to phosphoric(V) acid and phosphoric(V) acid and copper (II) nitrate according to the scheme that is presented in Figure 15. Phosphorus and copper were subsequently determined by means of Flame Atomic Absorption Spectroscopy (FAAS) spectrophotometry (determination of copper) and Inductively Coupled Plasma Mass Spectrometry (ICP-MS) (the determination of phosphorus). 

### 2.9. Flame Atomic Absorption Spectroscopy FAAS

The determination of copper content in samples Cell-*O*^6^-P(O)(OH)-H × Cu^2+^ (h) were assessed after prior digestion (Figure 15) by the FAAS method [71] and are listed in Table 9.

The results of determination of copper content in the phosphorylated cellulose samples illustrate the efficiency of the Cu-complexation reaction (-P(O)(OH)-H:Cu^2+^ = ca. 10:1). The results of FAAS analysis show that the copper concentation in the modyfied cellulose samples depends on the concentration of phosphite functions in the Cell-O^6^-P(O)(OH)-H, which increases with the duration of cellulose phosphorylation. Thus, samples with the higher content of cellulose phosphorus groups show the greater copper content after Cu-complexation reaction (Cell-*O*^6^-P(O)(O^−^)-H(6 h) × Cu^2+^: 263.8 mg/kg); Cell-*O*^6^-P(O)(O^−^)-H(48/72 h) × Cu^2+^: 659.2 and 655.4 respectively). There was no copper content in the cellulose sample Cell-*O*^6^-P(O)(OH)-H.

### 2.10. Specific Surface Area, Total Pore Volume and Average Pore Diameter Measurement

Table 10 presents the specific surface area S_BET_ [m^2^/g] measurements, obtained by the use of the BET technique [72], of the cellulose (determined and literature data), cellulose phosphates Cell-*O*^6^-P(O)(OH)-H and Cell-*O*^6^-P(O)(O^−^)-H(48 h) × Cu^2+^ complex. Several data on specific surface area of Avicel PH-101/102 obtained by use of the BET technique are reported in the literature [72,73,74,75,76,77]. The literature results of cellulose specific surface area are in a wide range from 1–5.7 [m^2^/g], determined using the nitrogen gas adsorption method [63,72,73,74,75,76,77] to 149–161 [m^2^/g] while using the water vapor adsorption method [73]. The large differences in BET results may be related to different types of samples pre-treatments [72,73].

The specific surface area of the unmodified cellulose (Avicel) is equal to 1,99 [m^2^/g] (Table 11). The phosphorylation of cellulose by PCl_3_ results in a gradual decrease of specific surface area from 1.99 [m^2^/g] to 1.11 [m^2^/g] for Cell-*O*^6^-P(O)(OH)-H(6 h) and, consequently, to 0.83 [m^2^/g] for Cell-*O*^6^-P(O)(OH)-H(72 h). This trend can be the result of substitution of the 6-hydroxyl function of cellulose (hydrogen bond acceptor and donor) by the multifunctional H-phosphonate function (P = O, P-O-H, P-O-C), which is able to form four hydrogen bonds with surrounding hydroxyls of the cellulose matrix. 

The phosphorylations of cellulose by PCl_3_ results in a gradual decrease of the specific surface area from 1.99 [m^2^/g] to 1.11 [m^2^/g] for Cell-*O*^6^-P(O)(OH)-H(6 h) and, consequently, to 0.83 [m^2^/g] for Cell-*O*^6^-P(O)(OH)-H(72 h). This trend can be the result of substitution 6-hydroxyl function of cellulose by the difunctional H-phosphonate function, which is able to form at least to two hydrogen bonds with surrounding hydroxyls of cellulose matrix. Therefore, the surface of Cell-*O*^6^-P(O)(OH)-H gradually rolls up with an increase of D_P_/D_S_ index. At the same time, complexation of phosphorylated cellulose (Cell-*O*^6^-P(O)(OH)-H(48 h)→Cell-*O*^6^-P(O)(O^−^)-H(48 h) × Cu^2+^) leads to a complex in which both the donor-acceptor of hydrogen bonds of H-phosphonate function are blocked by copper causing an increase of the specific surface area up to 1.75 [m^2^/g] in Cu-complex (Cell-*O*^6^-P(O)(OH)-H(48 h) × Cu^2+^) (see the structures in Figure 4).

Is worth to note, that in Oshima paper [77] the specific surface areas of cellulose adsorbents determined using the N_2_-BET method were 19.2 m^2^/g for phosphorylated bacterial cellulose (PBC), 2.4 m^2^/g for phosphorylated plant cellulose (PPC), whereas 27.3 m^2^/g for BC, and 1.0 m^2^/g for PC. 

**Figure 16:**
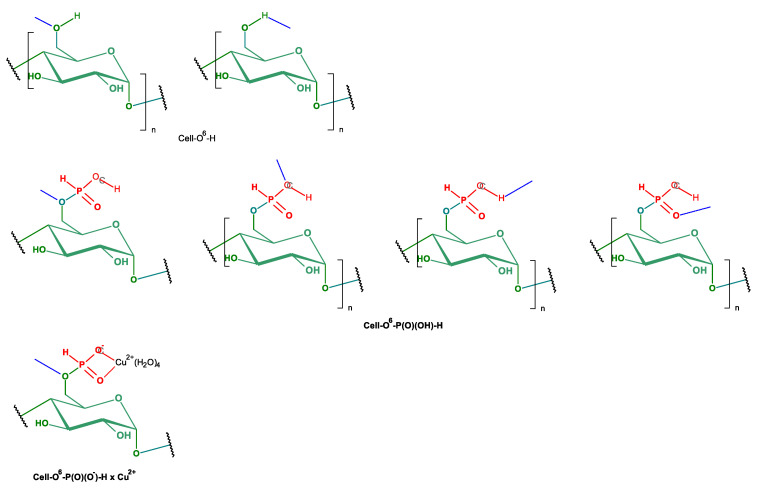
The ability for the formation of hydrogen bonds by 6-hydroxyl of cellulose (Cell-*O*^6^-H), cellulose-6-hydrogenphosphate (Cell-*O*^6^-P(O)(OH)-H) and cellulose-6-hydrogenphosphate-cooper (II) complex (Cell-*O*^6^-P(O)(OH)-H × Cu^2+^). Dotted lines in blue present possible hydrogen bonds with appropriate acceptors/donors.

Therefore, the surface of Cell-*O*^6^-P(O)(OH)-H gradually rolls up with the increase of D_S_ index; this increases with the phosphorylation time. At the same time, the complexation of phosphorylated cellulose (Cell-*O*^6^-P(O)(OH)-H(48 h)→Cell-*O*^6^-P(O)(OH)-H(48 h) × Cu^2+^) leads to a complex in which both donor-acceptor of hydrogen bonds of H-phosphonate function are blocked by copper causing increase of the specific surface area up to 1.75 [m^2^/g] in Cu-complex (Cell-*O*^6^-P(O)(O^−^)-H(48 h) × Cu^2+^) (see the structures in Figure 16).

### 2.11. Antibacterial Activity

**Figure 17:**
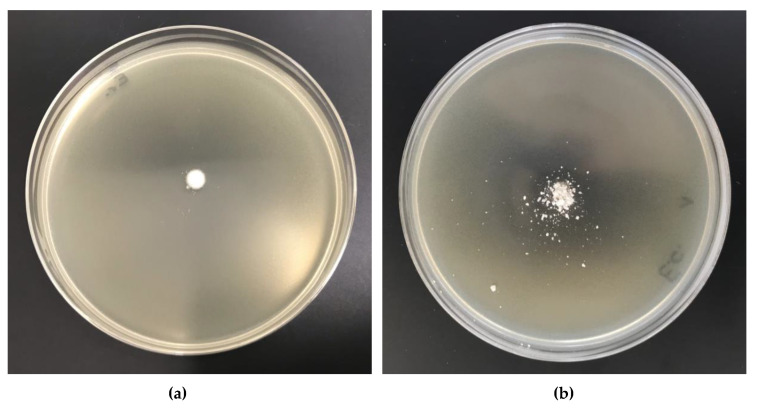
The Cell-*O*^6^-P(O)(O^−^)-H × Cu^2+^ complexes antimicrobial activity tests against *Escherichia* *Coli.* Inhibition zones of bacterial growth on Petri dishes: (**a**) Cell-*O*^6^-P(O)(OH)-H(48 h), (**b**) Cell-*O*^6^-P(O)(OH)-H(48 h) × Cu^2+^.

**Figure 18:**
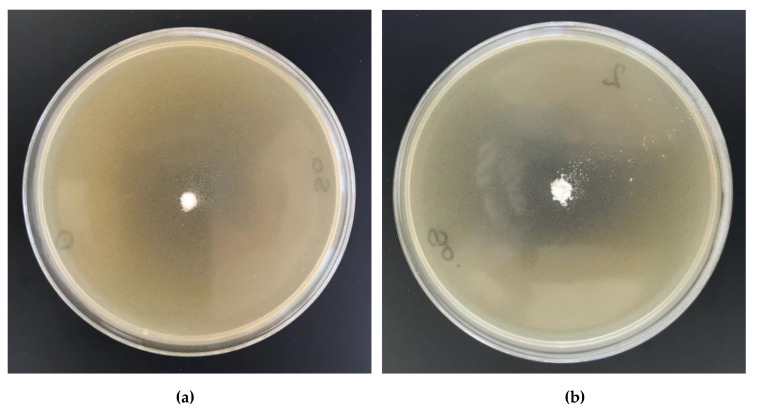
The Cell-*O*^6^-P(O)(O^−^)-H × Cu^2+^ complexes antimicrobial activity tests against *Staphylococcus aureus*. Inhibition zones of bacterial growth on Petri dishes: (**a**) Cell-*O*^6^-P(O)(OH)-H(48 h), (**b**) Cell-*O*^6^-P(O)(OH)-H(48 h) × Cu^2+^.

All of the synthesized cellulosic complexes were tested for their antimicrobial activities, in which *Escherichia Coli* (Gram-negative bacteria, ATCC11229) and *Staphylococcus aureus* (Gram-positive bacteria, ATCC 6538) were adopted as the bacterium models. Their antibacterial activities were determined with the agar plate diffusion method. Table 11 lists the results of antibacterial activity tests and Figure 17 and Figure 18 illustrate the bacterial growth on Petri dishes.

The results of tests on the antibacterial activity of Cell-*O*^6^-P(O)(OH)-H × Cu^2+^ composites, according to standard EN-ISO 20645:2006 [78].

Table 12 summarizes the antibacterial properties of various metal salts/nanoparticles and antibiotics against representative gram positive (*Escherichia coli*) and gram negative (*Staphylococcus aureus*) bacteria.

Some recent papers have described similar results [81,82,83,84].

**Figure 19:**
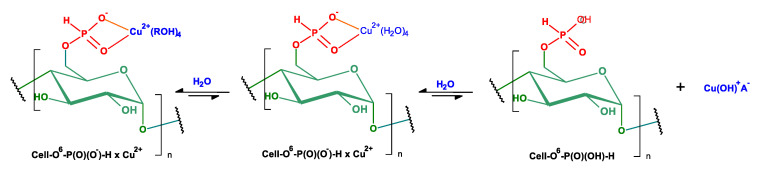
Release of copper ions from composite Cell-*O*^6^-P(O)(O^−^)-H × Cu^2+^ (A—anion derived from agar gel buffer).

Lower ZOI values of the composites Cell-*O*^6^-P(O)(O^−^)-H × Cu^2+^ in comparison with ZOI of soluble copper salts/nanoparticles is caused by a strong binding of copper ions by the functionalities of Cell-*O*^6^-P(O)(OH)-H, namely by hydrogen-phosphate (III) function, and also by surrounding cellulose hydroxyls. This results in a slow release of copper from the surface of composite, presumably driven by a hydrolysis [85,86,87], which limits a concentration of unbounded Cu (II) cations (Figure 19). 

The results of biological studies prove antimicrobial protection against different: Gram-negative (*Escherichia coli)* and Gram-positive (*Staphylococcus aureus)* bacterial microorganisms of biofunctionalized cellulose materials, expressed by visible inhibition zones of bacterial growth on Petri dishes and no visible bacterial growth under the samples (50× microscope magnification). Copper content concentrations of approximately 650–660 mg/kg in modified cellulose samples (Cell-*O*^6^-P(O)(OH)-H(48 h) × Cu^2+^, Cell-*O*^6^-P(O)(OH)-H(72 h) × Cu^2+^) provide antimicrobial properties according to the EN-ISO 20645:2006 standard (Table 11, Figure 17 and Figure 18) [78].

## 3. Materials and Methods 

### 3.1. Materials

Table 13 lists the reagents and standard solutions applied. All of these materials and solvents were used as received without further purification and were purchased from Merck (Darmstadt, Germany). Double distilled water was used in all of the experiments. Bacterial strains: *Escherichia coli* (ATCC 25922) and *Staphylococcus aureus* (ATCC 6538) were purchased from Microbiologics (St. Cloud, MA, USA).

### 3.2. Methods

#### 3.2.1. Specific Surface Area

The specific surface area of the investigated samples was measured using the Autosorb-1 (Quantachrome Instruments, Boynton Beach, FL, USA) apparatus. The analysis was performed while using the physisorption method with nitrogen being used as a sorption agent [72]. The measurements were carried out at 77 K. For each experiment, about 1 g of a given sample was weighed and used. Prior to the analysis, the samples were dried in 105 °C for 24 h and then degassed overnight at room temperature. 

The five-point Brunauer–Emmett–Teller (BET) method was applied in order to determine the specific surface area. The specific surface area was calculated twice for each sample, using the five-point adsorption isotherm (P/P_0_ in the range of 0.10–0.30) and the 39–point adsorption-desorption isotherm. 

#### 3.2.2. SEM/EDS—Scanning Electron Microscopy/Energy-Dispersive X-ray Spectroscopy

The microscopic analysis of samples was performed on a Tescan Vega 3 scanning electron microscope (Brno, Czech Republic) with the EDS Oxford Instruments (Abingdon, UK) X-ray micro analyzer. SEM microscopic examination of the surface topography was performed under high vacuum using the 20 ekV probe beam energy. The surface of each preparation was sprayed with a conductive substance (gold), while using a vacuum dust extractor (Quorum Technologies Ltd., Lewes, UK). The magnification was from 500× to 20000×.

#### 3.2.3. ATR-FTIR—Attenuated Total Reflection Fourier Transform Infrared Spectroscopy

The chemical structure of cellulose samples surface was assessed using ATR-FTIR spectroscopy in the range of 400–4000 cm^−1^ using a spectrometer Jasco’s 4200 (Tokyo, Japan) with an ATR attachment Pike Gladi ATR (Cottonwood, AZ, USA).

#### 3.2.4. Potentiometric Titration of Cell-*O*^6^-P(O)(OH)-H

Potentiometric titrations were performed using a Cerko-Lab System (Gdynia, Poland) microtitrator that was equipped with a combined glass electrode Hydromet ERH-13-6 (Gliwice, Poland). Cell-*O*^6^-P(O)(OH)-H (5 mg) samples were placed into glass vessel, followed by an addition of water (2 mL). Subsequently, under intensive stirring, the suspensions were titrated with KOH (0.016 M, carbonate-free), under inert atmosphere (Ar bubbling), at room temperature in the pH range of 2–12. Each titration was repeated at least four times. 

#### 3.2.5. ICP-MS—Inductively Coupled Plasma Mass Spectrometry—Determination of Phosphorus by Means of Inductively Coupled Plasma Mass Spectrometry

The method consists of the degradation of cellulose-*O*-phosphate (P(III)) to phosphoric acid (P(V)) (Figure 15) and the subsequent analysis of the obtained solution using the ICP-MS technique. Degradation/digestion of the sample was carried out in the mixture: nitric acid, hydrogen peroxide, water, and accelerated by ultrasound irradiation (temperature 200 °C, microwave digestion, 15 min.).

The decomposition of samples was carried out in a computer-controlled, closed, single-module microwave mineralizer Magnum II (Ertec, Wrocław, Poland), which was equipped with an integrated pressure temperature control. The process was performed by the wet method, in a closed single-module vessel with a 110 mL reaction chamber under elevated pressure. Microwave energy accelerated the degradation processes. The microwaves were absorbed by the reagents (usually acid or salt solutions) resulting in an increase of temperature and pressure, so that the mushroom-shaped membrane rose, and five heads appeared to accelerate the rapid decomposition of the sample or its chemical synthesis.

Elemental analyses (C and H) were recorded on an Elemental Analyzer Euro EA (Eurovector, Pavia, Italy), phosphorus determinations were performed after prior digestion of cellulose phosphate samples, while using an Agilent 7900 ICP-MS Spectrometer (Santa Clara, CA, USA) that was equipped with a quadruple mass analyzer. 

#### 3.2.6. Degradation of Cell-*O*^6^-P(O)(OH)-H

Sample Cell-*O*^6^-P(O)(OH)-H (0.03 ± 0.0001 g) was transferred into a reaction vessel of a mineralizer containing a degradation solution, which consisted of a mixture of HNO_3_ (67%, 1 mL), H_2_O_2_ (30%, 1 mL), and water (4 mL). The vessel was locked and placed into a mineralizer (degradation parameters: temperature 180–200 °C (±10 °C), max. pressure 20 bar, 15 min, power 100%). After degradation digest (Figure 15a) was quantitatively transferred into volumetric flask and diluted to 50 mL by water.

#### 3.2.7. FAAS—Atomic Absorption Spectrometry with Flame Excitation 

The determination of copper content in Cell-*O*^6^-P(O)(OH)-H(t) × Cu^2^^+^ samples was assessed using single-module Magnum II microwave mineralizer from Ertec (Wroclaw, Poland) and Thermo Scientific Thermo Solar M6 (LabWrench, Midland, MD, Canada) atomic absorption spectrometer.

The total copper content of the sample M [mg/kg; ppm] was calculated according to the formula [71]:

(2) $$M\;=\;\frac{Ci\;\times V}{mi}\left[\frac{mg}{kg}\right]$$

where:

*C*—metal concentration in the tested solution [mg/L];

*m*—mass of the mineralized sample [g]; and

*V*—volume of the sample solution [mL].

#### 3.2.8. Microbial Activity

The antibacterial activity of Cu-cellulose biochelates was tested according to EN ISO 20645:2006 *Textile fabrics—Determination of antibacterial activity—Agar diffusion plate test* [78] against a colony of gram-negative bacteria: *Escherchia coli* (ATCC 25922) and gram-positive bacteria: *Staphylococcus aureus* (ATCC 6538), analogously as polypropylene nonwovens [37].

The antibacterial activity of samples was tested by the agar diffusion method using Muller–Hinton medium agar. The test was initiated by pouring each agar onto sterilized Petri dishes and it was allowed to solidify. The surfaces of agar media were inoculated by overnight broth cultures of bacteria (ATCC 25922: 1.2 × 10^8^ CFU/mL, ATCC 6538: 1.7 × 10^8^ CFU/mL). Samples of the cellulose: phosphorylated derivatives and cellulose Cu-complex (Cell-*O*^6^-P(O)(OH)-H(48 h) × Cu^2+^) were placed onto the inoculated agar and then incubated at 37 °C for 24 h. The diameter of the clear zone around the sample was measured as an indication of inhibition of the microbial species. All of the tests were carried out in duplicate. Simultaneously, the same tests were carried out for control samples—samples of unmodified cellulose.

## 4. Conclusions

Cellulose phosphorylation in vapor phase with PCl_3_ has been completed after 48 h and afforded cellulose-*O*-phosphates (III) Cell-*O*-P(O)(OH)-H with substitution degree DS = 0.018.

The reaction (Cell-OH→Cell-*O*-P(O)(OH)-H) was carried out without solvent and co-reagents, in ambient temperature, in eco-friendly conditions. 

Cellulose-*O*-phosphates (III) Cell-*O*-P(O)(OH)-H, so obtained, have been converted into copper complex (Cell-*O*-P(O)(OH)-H→Cell-*O*^6^-P(O)(OH)-H × Cu^2+^).

All of the synthesized Cell-*O*-P(O)(OH)-H and Cell-*O*^6^-P(O)(OH)-H × Cu^2+^ samples have been analyzed while using an array of physical methods, including IR (confirmed the presence of phosphonate function) and NMR spectrometry (^31^P-NMR confirmed the selective O^6^-phosphorylation of AGU, it means the structure Cell-*O*^6^-P(O)(OH)-H), alkacymetric titration of acidic functionalities of the composite (confirmed one deflection point – characteristic for R-O-P(O)(OH)-H)), elemental analysis of composites (carbon and hydrogen—combustion analysis, phosphorus—IP AAS and copper—FAAS), investigations of Cell-*O*^6^-P(O)(OH)-H and Cell-*O*^6^-P(O)(OH)-H × Cu^2+^ morphology (SEM) and their specific surface activity.

For Cell-*O*^6^-P(O)(OH)-H(48 h), Cell-*O*^6^-P(O)(OH)-H(72 h), and Cell-*O*^6^-P(O)(OH)-H(48 h) × Cu^2+^, antibacterial tests against *Escherichia coli* (G-) and *Staphyloccoccus aureus* (G+) have been carried out in vitro (agar disc diffusion method). The determined antimicrobial properties of Cell-*O*^6^-P(O)(OH)-H × Cu^2+^ complexes revealed the antibacterial in vitro action against representative Gram-negative and Gram-positive bacteria.

For all of the synthesized composites Cell-*O*^6^-P(O)(OH)-H and Cell-*O*^6^-P(O)(OH)-H × Cu^2+^, we proposed the abbreviations system, coherent and compatible with earlier codes for functionalized alkane phosphonic acids and derivatives [35,36].

## Figures and Tables

**Table 1:** Characteristics of phosphorylation procedures afforded cellulose-*O*-phosphates (III).

No.	Reaction Conditions				Phosphorylation		Ref.
	Reagents ^a^	Reagent Ratio	Temp. [°C]	Time [h]	%	DS	
1	Cell-OH/Ar-O-PCl_2_	1:3	90	6		0.23	[29]
2	Cell-OH, NaOH, H_2_O/ Py, Ph-PCl_2_	1:11.6 and/or	100	1	0.78	0.04	[30]
	Cell-OH, NaOH, H_2_O/ Py, Ph-O-PCl_2_	1:10	100	1	0.23	0.01	
3	Cell-OH/H_3_PO_3_/urea or Cell-OH/H_3_PO_3_/urea (DMF)	1:10:16.5	150	0.5–8.0		0.6–2.0	[31]
		1:2.7:5.5 ^(DMF)^	150	0.5–8.0			
		1:10:17	150	2–5	12–13	0.96–1.0	[32]
		1:10:17	150	4	12.6	1.01	
					8.6 *	0.62 *	
		X:1:15	150	1–9	9.2–21.4	0.6–2.0	[33]
4	Cell-OH/H_3_PO_3_/urea/ MW	1:10:16	85 ^(MW)^	6		0.6	[34]
		1:10:16	105 ^(MW)^	2		2.8	

^a^ Reagents were calculated based on the anhydroglucose unit [AGU, M = 162]. Abbreviations: Py—pyridine; DMF—dimethyl formamide; DS—substitution/(phosphorylation) degree. * After diafiltration. MW—microwave irradiation.

**Table 2:** Cellulose complexing reaction solution.

Reagent		Reaction Time
Cu(NO_3_)_2_ in HNO_3_	0.1 [mL] ^/a^	
H_2_O	0.5 [mL]	
Cell-*O*^6^-P(O)(OH)-H	50 [mg]	2 [h]

^/a^ Copper(II) nitrate [Cu(NO_3_)_2_ (1000 mg Cu/L; 15.7 mmol Cu/L) in 0.5 M HNO_3_].

**Table 3:** Solubility of Cell-*O*^6^-P(O)(OH)-H in aqueous and ionic liquids solutions.

No.	Cell-*O*^6^-P(O)(OH)-H	Solution Components				Temp. [°C]	Time [h]	Solub.
		Solvent 1	mL	Solvent 2	mL			
1	25 mg	H_2_O	2	−	−	25	2	−
2	25 mg	H_2_O	2	−	−	50	2	−
3	25 mg	0.1 M KOH	2	−	−	25	2	−
4	25 mg	0.1 M KOH	2	−	−	50	2	−
5	25 mg	TBAA	0.2 (g)	DMSO	0.72 mL; (0.8 g)	25	2	+/−
6	25 mg	TBAA	0.2 (g)	DMSO	0.72 mL; (0.8 g)	50	2	++/−

TBAA—tetrabutylamonium acetate; DMSO—dimethyl sulfoxide; Solubility (solub.): (−) not soluble; (+/−)—partially soluble; (++/−)—soluble with tiny suspension.

**Table 5:** IR of cellulose-*O*-phosphates.

Cell-*O*-P(O)(H)(OH)		Cell-*O*-P(O)(OH)_2_				Cell-*O*-P(O) (OH)(O-Ph)	Vibration Mode
[28]	[27]	[68]	[28]	[24]	[11]	[28]	
3460	3400–3500	3400	3460	2990–3630	3402	3460	OH
2940; 1430	2800–2900	2920	2940; 1430	2891	2891	2940; 1430	CH, CH_2_
	1160; 1120			2360			C-O-C
2320	2370			1383			P–H
1650		1640–1660	1650		1625	1650	H-OH
					1418; 1382; 1152; 1029		C-O
1250	1210	1250–1300		920–1000	1383	1370	P=O
	920–1000	1395	1000–1400				P-OH
1000–1060	810				^1^	1040–1190	P-O-C
1075			1075			1075	C-OH
905–910		700–100	905–910			905–910	pyranose ring
			520–600				P(O)-H

**Table 7:** Representative methods of potentiometric titration of cellulose-*O*-phosphoric acids.

Cellulose- Phosphates	Potentiometric Titrations		Ref.
	Mode of Titration	Defl. Points	
**Cell-*O*-P(O)(OH)-H**	Direct titrations with KOH or LiOH	1	[31,32]
**Cell-*O*-P(O)(OH)_2_**	Direct titrations with LiOH, NaOH, KOH or Ba(OH)_2_	2	[18,27,30] ^/a^
	Reverse titration using KOH/HCl	1	[41,70]

^/a^ Defl. points—number of deflection points of the titration curve.

**Table 8:** The results of elemental analysis of Cell-O^6^-P(O)(OH)-H samples.

Samples ^/a^	Element Analysis ^/b^					DP ^/c^
	EA		ICP MS P			
	C [%]	H [%]	mg/kg	g/100 g [%]	mM/kg	
Cellulose	44.35 ± 0.04	6.22 ± 0.04	0	0	0	0
Cell-*O*^6^-P(O)(OH)-H (6 h)	41.25 ± 0.04	6.20 ± 0.02	1381 ± 12	0.1381	44.5	0.007
Cell-*O*^6^-P(O)(OH)-H (24 h)	41.21 ± 0.04	6.23 ± 0.03	2872 ± 55	0.2872	92.6	0.015
Cell-*O*^6^-P(O)(OH)-H (48 h)	41.18 ± 0.06	6.15 ± 0.03	3537 ± 16	0.3537	114.1	0.019
Cell-*O*^6^-P(O)(OH)-H (72 h)	40.98 ± 0.06	6.21 ± 0.06	3416 ± 20	0.3416	110.2	0.018

^/a^ Cell-*O*^6^-P(O)(OH)-H(t[h]) concerns samples obtained by PCl_3_ phosphorylation of cellulose, carried out in “t” (h) time and after subsequent hydrolytic treatment (Cell-OH→Cell-*O*^6^-PCl_2_(t)→Cell-*O*^6^-P(O)(OH)-H(t)). ^/^^b^ Element Analysis data (average from duplicate) based on: CEA—Combustion Elemental Analysis data (C & H); ICP-MS—Inductively-Coupled-Plasma Mass Spectrometry data (P). ^/c^ DP—based on the ICP MS determinations, calculated according to Equation (1) [33].

**Table 9:** Copper content in Cell-*O*^6^-P(O)(OH)-H samples and their copper complexes Cell-*O*^6^-P(O)(O^−^)-H × Cu^2+^.

No.	Sample ^/a^	Phosphorylation Time [h]	Cu Concentration		
			mg/kg ^/b^	g/100 g [%]	mMol/ kg
1	Cell-O^6^-P(O)(OH)-H	0	0	0	0
2	Cell-*O*^6^-P(O)(O^−^)-H (6 h) × Cu^2+^	6	263.8	0.0264	4.15
3	Cell-*O*^6^-P(O)(O^−^)-H (24 h) × Cu^2+^	24	423.6	0.0424	6.67
4	Cell-*O*^6^-P(O)(O^−^)-H (48 h) × Cu^2+^	48	659.2	0.0659	10.37
5	Cell-*O*^6^-P(O)(O^−^)-H (72 h) × Cu^2+^	72	655.4	0.0655	10.32

^/a^ Cell-*O*^6^-P(O)(OH)-H(t) obtained after given time of cellulose phosphorylation. ^b/^ The results have been measured in triplicate and presented as mean value with deviation approximately ±2%.

**Table 10:** Specific surface area (S_BET_) determinations of the examined samples.

No.	Cellulose, Cell-*O*^6^-P(O)(OH)-H and Cell-*O*^6^-P(O)(O^−^)-H(48 h) × Cu^2+^	Specific Surface Area ^/a^ S_BET_ [m^2^/g]		Ref.
		N_2_-BET Method	H_2_O_(gas)_-BET Method	
1	Microcrystalline cellulose	1	149	[73]
		1.2	161	
2	Avicel PH 102 MCC powder	1.3		[74]
3	Cellulose linters	2.8		[63]
	Cellulose mercerized linters	1.0		
4	Avicel CE 15	0.5		[75]
	Avicel DG	1.2		
	Avicel HFE 102	0.6		
5	Avicel PH-101	5.71		[76]
	Avicel PH-101 (ball milled)	0.87		
6	Cellulose Avicel PH-101	1.99		This work
	Cell-O^6^-P(O)(OH)-H (6 h)	1.11		
	Cell-O^6^-P(O)(OH)-H (24 h)	0.88		
	Cell-O^6^-P(O)(OH)-H (48 h)	0.88		
	Cell-O^6^-P(O)(OH)-H (72 h)	0.83		
	Cell-O^6^-P(O)(O^−^)-H(48 h)×Cu^2+^	1.75		

^/a^ N_2_-BET method–determined by N_2_ gas adsorption. H_2_O_(gas)_-BET method–determined by H_2_O gas adsorption.

**Table 11:** Results of tests on the antibacterial activity of Cell-*O*^6^-P(O)(O^−^)-H × Cu^2+^ complexes, according to standard EN-ISO 20645:2006 [78].

No.	Cell-*O*^6^-P(O)(OH)-H (t) ^/a^	Cu in Cell-*O*^6^-P(O)(O^−^) × Cu^2+^)-H			ZOI ^/b^ [mm]	
		In The Starting Composite	Spots Deposited		Bacteria Average ^/c^	
		mg/ kg ^/d^	μg/ disc ^/a^	μMol/ disc ^/a^	*E.c.*	*S.a.*
1	Cell-*O*^6^-P(O)(OH)-H	0	0	0	-	-
2	Cell-*O*^6^-P(O)(OH)-H (6 h) × Cu^2+^	263.8	0.26	0.04	-	-
3	Cell-*O*^6^-P(O)(OH)-H (24 h) × Cu^2+^	423.6	0.42	0.07	-	-
4	Cell-*O*^6^-P(O)(OH)-H (48 h) × Cu^2+^	659.2	0.66	0.10	1	1
5	Cell-*O*^6^-P(O)(OH)-H (72 h) × Cu^2+^	655.4	0.66	0.10	1	1

^/a^ 10 mg of composite Cell-*O*^6^-P(O)(O^−^)-H × Cu^2+^ was used for preparation of the disc. ^/b^ Zone of inhibition. ^/c^ Concentration of inoculum (bacterial suspension) amount of live bacteria: *Escherichia coli*: CFU/mL = 1.2 × 10^8^; *Staphylococcus aureus*: CFU/mL = 1.7 × 10^8^. ^/d^ Values of Table 9.

**Table 12:** Antibacterial properties of various metal salts/nanoparticles and antibiotics against representative gram positive (*Escherichia coli*) and gram negative (*Staphylococcus aureus*) bacteria, reflexed by their zone of growth inhibition (ZOI).

No	Antibacterial Agent	Agent Deposited On Spot		ZOI [mm]		Lit.
		mg/spot	μmol/spot	*Escherichia coli*	*Staphylococcus aureus*	
1.1	CuCl_2_		0.05	15	14	[79]
1.2	AgNO_3_		0.05	16	15	
2.3	CuNPS ^/a^		0.05	17	16	
3.4	Gentamycin	0.01	0.02	19	13	
1.5	Penicillin	0.01	0.03	0	17	
1.6	Tetracycline	0.03	0.07	19	19	
2.1	CuSO_4_	0.06	0.38	0	0	[80]
		0.12	0.75	0	0	
		0.24	1.5	9.4	8.2	
		1.92	12	13	14	
2.2	Oxytetracycline	1.80	3.9	23	23	
2.3	CNPs ^/b^	0.06	0.9	15	14	
		0.12	1.8	17	20	
		0.24	3.6	23	22	
		1.92	30.	38	37	

^/a^ Synthesized by reduction of CuCl_2_ by ascorbic acid. ^b/^ Synthesized by reduction of CuSO_4_ by hydrazine.

**Table 13:** Materials and reagents.

Nr.	Name	CAS
1	Cellulose microcrystalline (Avicel PH-101), ~50 μm particle size	9004–34–6
2	Phosphorus trichloride, 99%	7719–12–2
3	D-Glucose 6-phosphate disodium salt hydrate, ≥98%	3671–99–6
4	Nitric acid, 65%, Suprapur^®^	7697–37–2
5	Hydrogen peroxide 29.0–32.0%	7722–84–1
6	Copper(II) nitrate [Cu(NO_3_)_2_ (1000 mg/L Cu) in 0.5 M HNO_3_	13778–31–9

## Data Availability

Data is contained within the article or Supplementary Material.

## Supplementary Materials

The following are available online at https://www.mdpi.com/2079-6382/10/2/203/s1.
