# Supplementary material for: Vapor Phosphorylation of Cellulose by Phosphorus Trichlo-Ride: Selective Phosphorylation of 6-Hydroxyl Function—The Synthesis of New Antimicrobial Cellulose 6-Phosphate(III)-Copper Complexes
Source: Antibiotics (Basel). 2021 Feb 19;10(2):203. doi: 10.3390/antibiotics10020203 (PMC7923017; doi:10.3390/antibiotics10020203)
Supplement: Supplementary file 1 [file antibiotics-10-00203-s001.pdf]

antibiotics

MDPI

Supplementary Materials

Vapor Phosphorylation of Cellulose by Phosphorus Trichlo-Ride: Selective Phosphorylation of 6-Hydroxyl Function. The Synthesis of New Antimicrobial Cellulose 6-Phosphate(III)-Copper Complexes.

Marcin H. Kudzin[1], Zdzisława Mrozińska[1] and Paweł Urbaniak[2]

[1] Łukasiewicz Research Network—Textile Research Institute, Brzezinska 5/15, Lodz 92-103, Poland; zmrozinska@iw.lodz.pl

[2] Faculty of Chemistry, University of Lodz, Tamka 12, Lodz 90-136, Poland; pawel.urbaniak@chemia.uni.lodz.pl

1. Phosphorylation of cellulose

The phosphorylation reactions of the cellulose in the exposure of phosphorus trichloride (PCl_3) were carried out in the set consisting of two glass weighing bottles: the larger one (D vs. H: 40 mm × 40 mm), and the inner vessel (D vs. H: 20 mm × 20 mm). A 0.05 g portion of cellulose was poured into the inner vessel (Figure. 1a). Raschig rings were placed in the larger bottle (h = 1 cm), then PCl_3 (1 ml) was added (Figure. 1b), followed by placing the inner vessel (with cellulose) into the bottle with PCl_3, followed and the whole closing with a lid (Figure. 1c).

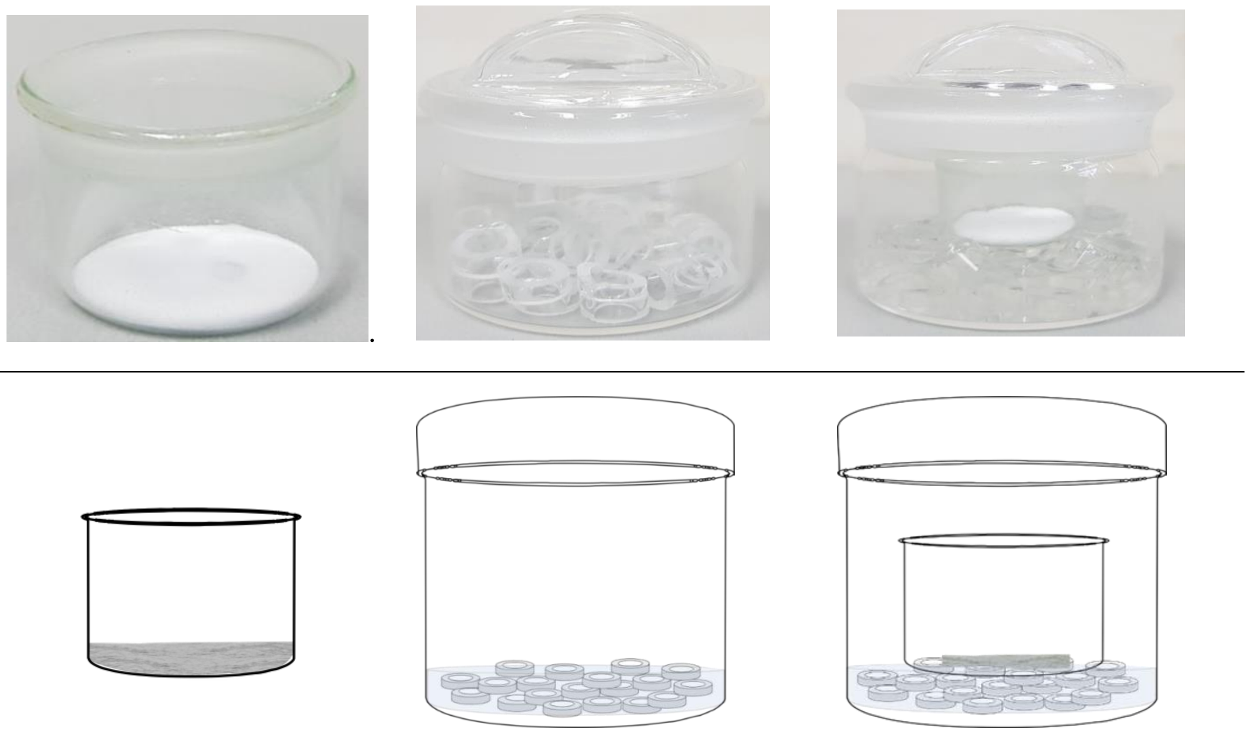

(a). **(b)** **(c)**

Figure S1. Assembly of the reactor for conducting phosphorylation reaction with PCl_3 vapors: (**a**) inner vessel with cellulose sample, (**b**) bottle with PCl_3, (**c**) phosphorylation reaction reactor.

2. Antimicrobial Activity

The antibacterial activity of the Cell-O^6-P(O)(O-)-H × Cu^{2+} was tested according to EN ISO 20645:2006. *Textile Fabrics. Determination of Antibacterial Activity—Agar Diffusion Plate Test* [78]. Gram-negative bacteria *E. coli* (ATCC 25922) and Gram-positive bacteria *S. aureus* (ATCC 6538) were used as model bacteria in this study. Antibacterial activity of Cell-O^6-P(O)(O-)-H × Cu^{2+} complex was tested by the agar diffusion method using Muller Hinton medium agar. The test was initiated by pouring each agar onto sterilized Petri dishes and allowing it to solidify. The surface of the growth media was inoculated by overnight broth cultures of bacteria (0.4 × 108 CFU mL^{-1}). Samples of modified cellulose were placed onto the inoculated agar and incubated at 37°C for 24 h. The diameter of the clear zone around the sample was measured as an indication of growth inhibition. All tests were carried out in duplicate. Simultaneously, the same tests were carried out as control samples with samples of unmodified cellulose or Cell-O^6-P(O)(O-)-H.
